# Charge Density Study of Two-Electron Four-Center Bonding in a Dimer of Tetracyanoethylene Radical Anions as a Benchmark for Two-Electron Multicenter Bonding

**DOI:** 10.1021/acs.cgd.4c00342

**Published:** 2024-07-22

**Authors:** Miha Virant, Petar Štrbac, Anna Krawczuk, Valentina Milašinović, Petra Stanić, Matic Lozinšek, Krešimir Molčanov

**Affiliations:** † Jožef Stefan Institute, Jamova Cesta 39, SI-1000 Ljubljana, Slovenia; ‡ Rud̵er Bošković Institute, Bijenička 54, HR-10000 Zagreb, Croatia; § University of Göttingen, Tammanstrasse 4, D-37077 Gottingen, Germany

## Abstract

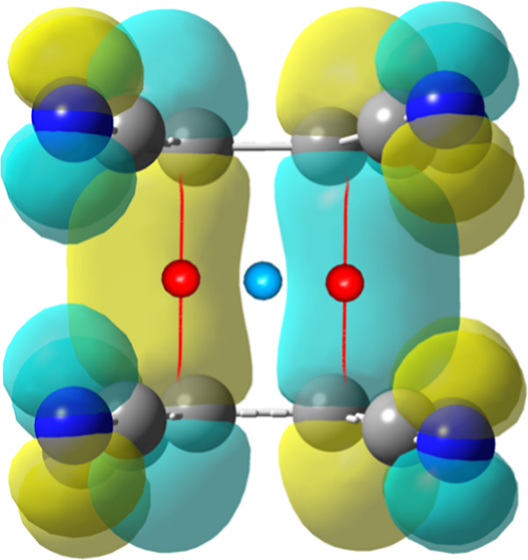

The dimer of the tetracyanoethylene (TCNE) radical anions represents the simplest and the best studied case of two-electron multicenter covalent bonding (2e/mc or *pancake bonding*). The model compound, *N*-methylpyridinium salt of TCNE^•–^, is diamagnetic, meaning that the electrons in two contiguous radicals are paired and occupy a HOMO orbital which spans two TCNE^•–^ radicals. Charge density in this system is studied as a benchmark for comparison of charge densities in other pancake-bonded radical systems. Two electrons from two contiguous radicals indeed form a bonding electron pair, which is distributed between two central ethylene groups in the dimer, i.e., between four carbon atoms. The topology of electron density reveals two bond critical points between the central ethylene groups in the dimer, with maximum electron density of 0.185 e Å^–3^; the corresponding theoretical value is 0.118 e Å^–3^.

## Abstract 2

A quantum crystallographic study of two-electron four-center bond, prototype of pancake bond between planar organic radicals, is studied in a model compound, *N*-methylpyridinium salt of tetracyanoethylene radical anion (TCNE^•−^) revealed that the two electrons from two contiguous radicals form a bonding electron pair, which is distributed between two central ethylene groups in the dimer, i.e., between four carbon atoms. The topology of electron density reveals two bond critical points between the central ethylene groups in the dimer, with maximum electron density of 0.185 e Å^−3^; the corresponding theoretical value is 0.118 e Å^−3^.

## Introduction

Bulk properties of organic radical-based materials are determined by coupling and decoupling of spins, which is in turn dependent on π-stacking and involves a considerable covalent component.^1,2^ This interaction, known as two-electron multicenter bonding (2e/mc or *pancake bonding*),^1−4^ is thus interesting not only from the fundamental (nature of chemical bonding and intermolecular interactions) but also from applicative point of view. Design of novel organic magnets and (semi)conductors therefore requires fine-tuning of this interaction.^5−12^

The covalent component implies pairing of spins of contiguous radicals, so that a HOMO orbital extends between both rings; the total interaction is rather strong and often exceeds −15 kcal mol^–1^.^4^ It also involves electrostatic, dispersion, and (local) dipolar components. Pancake bonding is thus one of the strongest intermolecular interactions, comparable to the strong hydrogen^13,14^ and halogen bonding,^15,16^ which are also partially covalent. The difference lies in the localization of the electron pair, which is not localized in the pancake bonding, but is rather distributed between two radicals, involving multiple centers.^1−4^

**Scheme 1:**
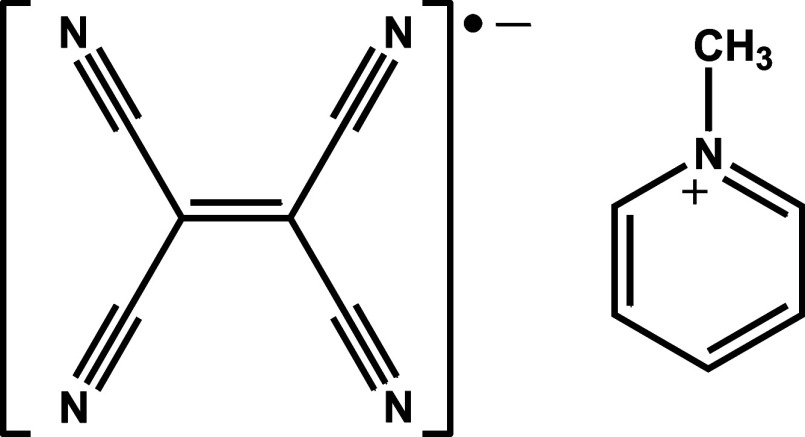
Molecular Scheme of Compound **1**: TCNE Radical Anion (Left) and *N*-Methylpyridinium Cation (Right)

**Figure 1:**
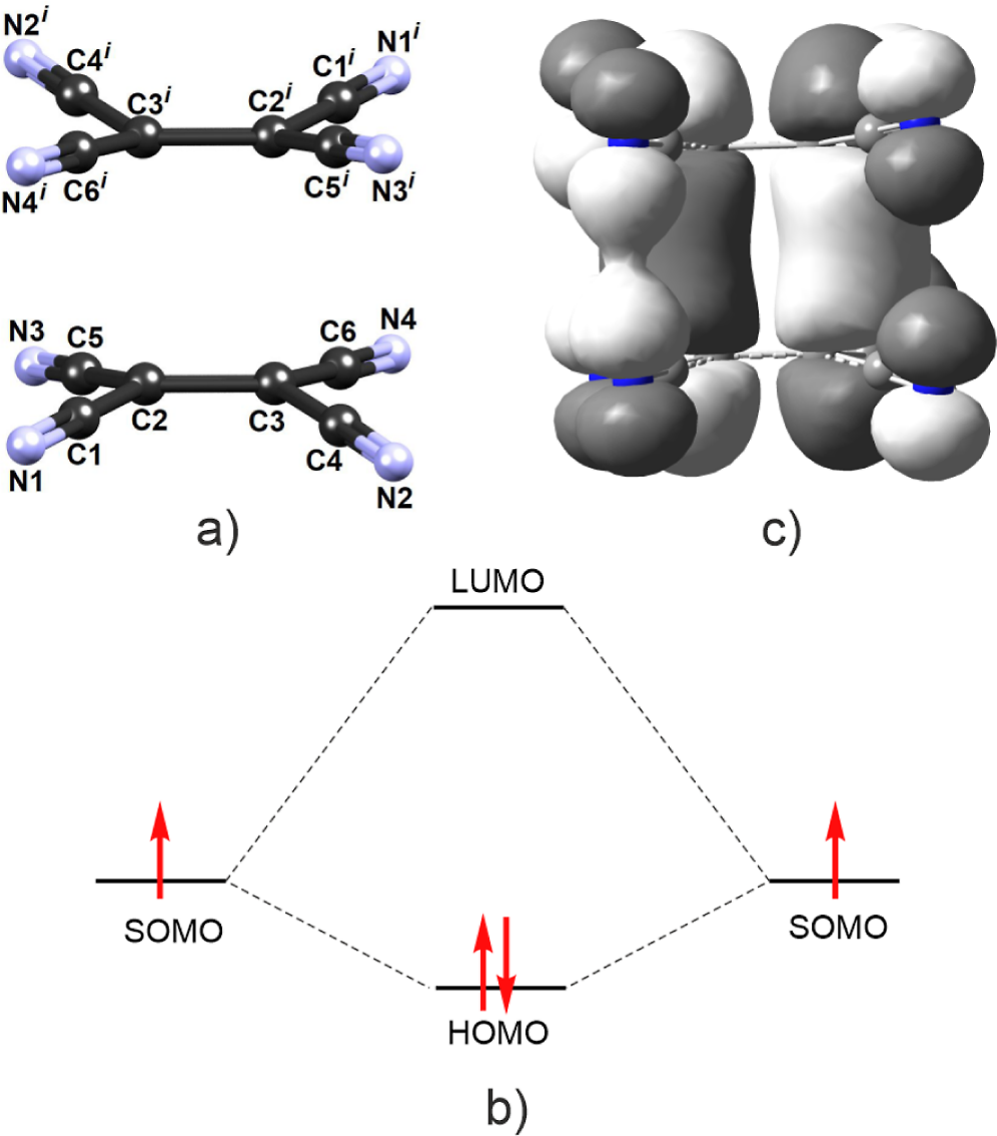
(a) Dimer of TCNE^•–^ radical anions in **1** with atom numbering scheme, (b) schematic representation of combination of two SOMO orbitals in two isolated TCNE^•–^ radicals into a HOMO orbital of a dimer (orbital levels correspond to energies obtained from DFT calculations for the studied compound), and (c) computed HOMO orbital of a dimer in **1**.

One of the simplest examples of pancake bonding (and due to small size of the moieties very convenient for the study of X-ray charge density) between two radicals is two-electron four-center (2e/4c) bond in dimers of tetracyanoethylene (TCNE^•–^, Scheme 1) radicals.^17−21^ It can be considered a model system for the study of interactions between the radicals. The central ethylene groups, C=C, form close contacts with intermolecular C···C distances of less than 2.89 Å. Systems involving this type of dimer are diamagnetic, so the interactions within a dimer have been interpreted as weak covalent π-bonding, and a computed HOMO orbital extends over both TCNE^•–^ radicals in a dimer (Figure 1). First prepared in 1958,^22^ TCNE is a well-known electron acceptor that is easily reduced into a radical anion. Two polymorphs of TCNE are known, monoclinic one,^23^ which has a partial orientational disorder of 4–5% of the molecules,^24^ and cubic, which is not disordered.^25^ The cubic phase was a subject of early charge density studies, by combined X-ray and neutron diffraction^26^ as well as theoretical methods.^27^

A survey of the Cambridge Structural Database (CSD)^28^ revealed 48 crystal structures with TCNE^•–^ radical anion. Six of these involve a disordered TCNE moiety; five involve a π-complex with a metal cation, and in one, the TCNE moieties are bound by a σ-bond. Of the remaining 36 structures (with 38 individual contacts), geometry consistent with 2e/mc bonding is found in 31 of them (85%), a longer contact with a large offset (interpreted as weak pancake bond^29^) is found in three structures, and two structures involve an isolated TCNE apparently forming no close contacts with other radical moieties. A full list of structures with CSD ref codes are given in Table S7. While the low number of structures and different temperatures of data collection (varying from 90 to 293 K; it has been shown that the length of pancake bonding is temperature-dependent^30,31^) preclude statistical analysis of geometry, some conclusions can be drawn, nevertheless. The closest distance between two C atoms, proposed by Miller and Novoa *et al*.^17b^ as a measure of close contact between two TCNE^•–^ radical anions, fall in the range 2.80–3.10 Å (Table S7). The radicals forming a 2e/4c bond are mostly parallel within the experimental error.

In this work, the first experimental charge density study of 2e/4c bonding in a dimer of TCNE^•–^ radicals, in its salt with *N*-methylpyridinium cation^32^ (**1**, Scheme 1) is presented. Since this compound comprises the simplest case of 2e/mc π-bonding and easily forms large well-developed single crystals,^32^ it can be used as a benchmark for comparison with other 2e/mc bonds in various radical systems. For a study of electronic structures of TCNE^•–^ radical, a comparison with a neutral TCNE molecule is important, so a redetermination of experimental charge density of cubic TCNE was also undertaken.

## Results and Discussion

### Structure and Charge of TCNE Radical Anion in **1**

The total fitted charge (derived from the refined *P*_val_ values) for the TCNE^•–^ anion is −0.925, and the theoretical one derived from generated structure factors via periodic density functional theory (DFT) computation is −0.970 (Table 1), which is close to the formal value of −1. Both models present similar trend, i.e., the majority of the negative charge is located on the two C atoms of the central ethylene fragment (C2 and C3) having partially negative charges, while the N and C atoms of the cyano groups are close to neutral.

**Table 1:** Atomic Charges Determined Based on Mutlipole Refinement on Experimental and Theoretical Structure Factors

	fitted charges	
	experimental	theoretical
N1	–0.057	–0.036
N2	+0.114	+0.018
N3	+0.058	–0.084
N4	–0.053	–0.036
C1	+0.054	+0.031
C2	–0.483	–0.496
C3	–0.483	–0.496
C4	–0.125	+0.014
C5	–0.007	+0.114
C6	+0.057	+0.001
**total TCNE**^**•–**^	**–0.925**	–**0.970**
N5	+0.636	+0.867
C7	–0.351	–0.781
C8	+0.129	+0.128
C9	–0.081	–0.202
C10	+0.136	+0.128
C11	–0.339	–0.781
C12	–1.237	–1.679
H7	+0.223	+0.538
H8	+0.091	+0.235
H9	+0.126	+0.270
H10	+0.092	+0.235
H11	+0.244	+0.538
H12A	+0.437	+0.501
H12B	+0.384	+0.501
H12C	+0.435	+0.501
**total cation**	**+0.925**	**+0.999**
neutral TCNE		
N1	+0.109	
C1	–0.130	
C2	+0.004	

The topology of the electron density in the TCNE^•–^ anion (Table 2) is compared with that of the neutral TCNE (Table 3). Without the negative charge, the topological order of the ethylene C2=C2^*i*^ [symmetry operator (*i*) −*x*, 1 – *y*, *z*] bond is 1.59, while the topological order of the single C–C bond (only one is symmetry-independent) is 0.97. In TCNE^•–^ anion, the bond orders of the formally single C–C bonds between the ethylene and cyano C atoms are 0.95–0.98. The central ethylene C2=C3 bond has a higher order (1.76) than that in the neutral molecule but with a lower electron density and a slightly less negative Laplacian (Tables 2 and 3). This is due to a HOMO orbital of a dimer (see below), which may be weakly antibonding for a single TCNE moiety. On the other hand, the topological parameters of the C≡N bonds (only one is symmetry-independent in the neutral TCNE) are very similar to those in the radical anion. This points out to the concentration of charge in the central ethylene fragment.

**Table 2:** Topology of the Experimental Electron Density Derived from Electron-Density (Regular Font) and Theoretical Electron Density (Italic) Derived after Multipole Refinement in a Dimer of TCNE^•–^ Radicals^a^

bond	length (Å)	electron density (e Å^–3^) ρ_cp_	Laplacian (e Å^–5^)	ellipticity	bond order *n*_topo_
N1–C1	1.1632(4)	2.882	–4.3	0.04	2.06
		*3.119*	*–13.29*	*0.01*	
N2–C4	1.1632(5)	3.014	–15.9	0.06	2.00
		*3.116*	*–12.77*	*0.01*	
N3–C5	1.1635(5)	3.014	–15.4	0.06	1.99
		*3.106*	*–11.69*	*0.01*	
N4–C6	1.1632(4)	2.858	–3.5	0.03	2.06
		*3.132*	*–14.18*	*0.02*	
C1–C2	1.4117(3)	1.808	–12.3	0.25	0.98
		*1.873*	*–12.72*	*0.18*	
C2–C3	1.4238(3)	1.889	–20.9	0.21	1.76
		*1.883*	*–13.42*	*0.27*	
C2–C5	1.4124(4)	1.794	–11.9	0.24	0.95
		*1.876*	*–12.94*	*0.18*	
C3–C4	1.4129(4)	1.818	–11.8	0.33	0.98
		*1.866*	*–12.56*	*0.18*	
C3–C6	1.4116(3)	1.807	–12.1	0.26	0.97
		*1.875*	*–12.68*	*0.17*	
C2–C3^*i*^	2.8087(3)	0.185	1.25	0.18	
		*0.118*	*1.15*	*0.04*	
C3–C2^*i*^	2.8087(3)	0.185	1.25	0.18	
		*0.117*	*1.15*	*0.04*	

a Symmetry operator (*i*) 1 – *x*, 1 – *y*, −*z*.

**Table 3:** Topology of Experimental Electron Density in Neutral TCNE, Derived from Multipole Refinement^a^

bond	length (Å)	electron density (e Å^–3^) ρ_cp_	Laplacian (e Å^–5^)	ellipticity	bond order *n*_topo_
N1–C1	1.15484(18)	3.374	–26.32	0.00	2.09
C1–C2	1.43098(13)	1.884	–13.62	0.08	0.97
C2–C2^*i*^	1.3626(3)	2.251	–21.74	0.34	1.59

a Symmetry operator (*i*) −*x*, 1 – *y*, *z*.

**Figure 2:**
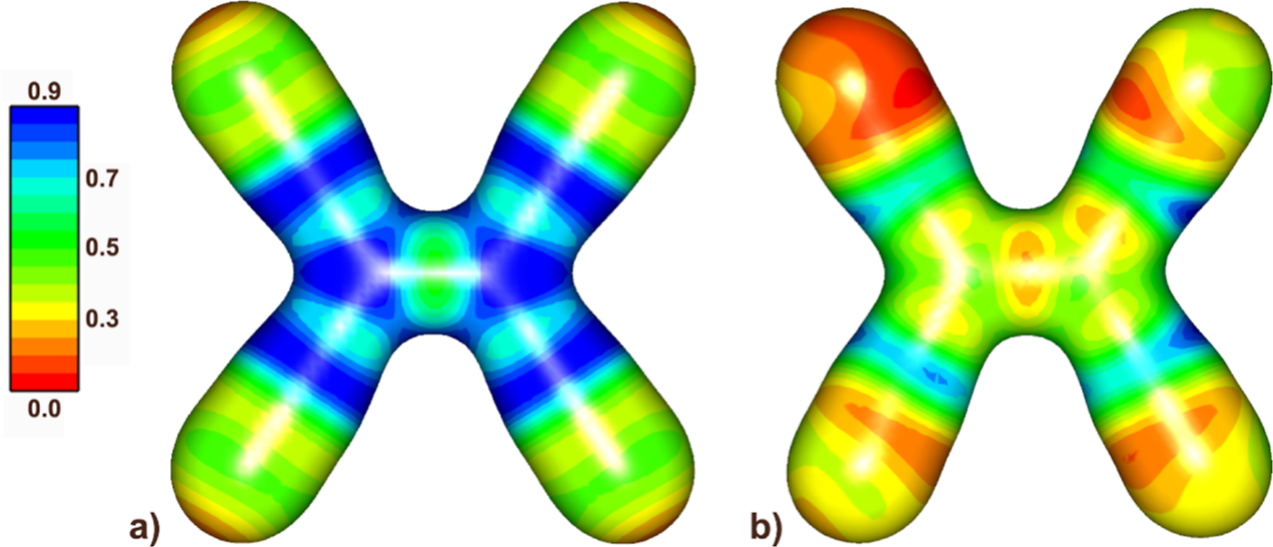
Electrostatic potential derived from the experimental multipolar model in (a) neutral TCNE and (b) TCNE^•–^ radical anion plotted onto an electron density isosurface of 0.5 e Å^–3^.

Fitted charges also show a delocalization of the negative charge (Table 1). The greatest difference is in the central ethylene atoms, −0.13 vs – 0.48. This accounts for 70% of the negative charge; the rest is distributed among the four cyano groups. The electrostatic potential is distributed more evenly (Figure 2), with regions of negative potential about the ethylene C=C bond and also around the cyano groups.

While the neutral TCNE is planar within the experimental error, a slight boat-like distortion of the TCNE^•–^ radical anion (Figure 1a) can be noted. It can be ascribed to the 2e/4c bonding in a dimer of radicals; the cyano-groups are bent out of the mean molecular plane to minimize repulsion and allow close contact of central C=C bonds. Thus, the mean molecular plane passes between the central C2=C3 bond and the four cyano-groups; the maximum deviations from the plane are −0.170 Å (for C3) and +0.188 Å (for N2).

The atomic charges and topological bond orders in the *N*-methylpyridinium cation (Table S1) are close to those found in its semiquinone salts^33^ and its related 4-cyano analogue.^34^

### Charge Density and QTAIM Study of Four-Center Two-Electron Bonding in **1**

**Figure 3:**
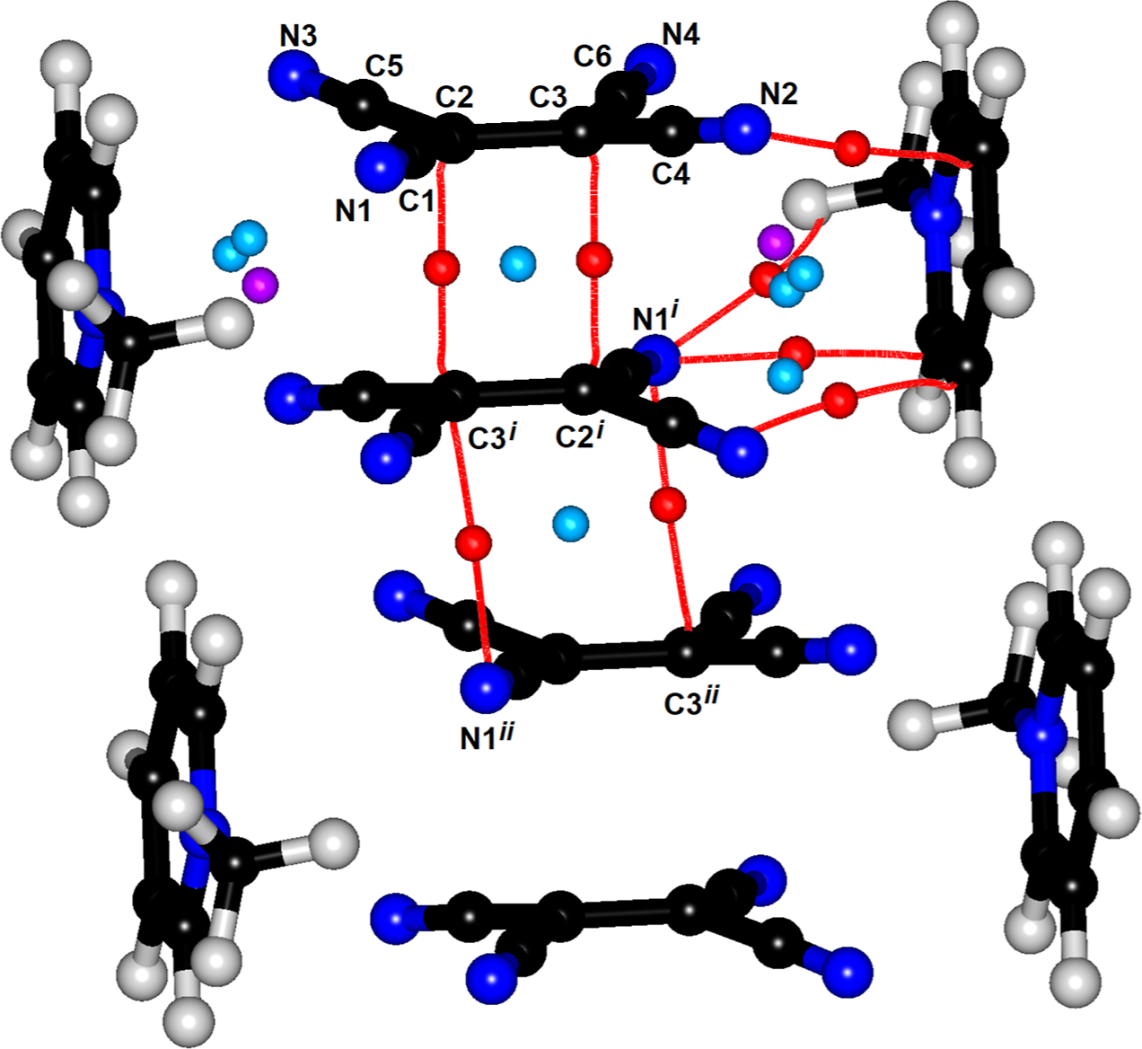
Experimentally determined critical points in two adjacent dimers of TCNE^•–^ radicals. (3, −1) cp’s are shown as red, (3, +1) as blue and (3, +3) as purple spheres. Bond paths are shown as red lines. Symmetry operators: (*i*) 1 – *x*, 1 – *y*, −*z*; (*ii*) 2 – *x*, 1 – *y*, −*z*.

Since the crystal structure of **1** was described before,^32^ only an outline will be provided here. Its packing (Figures 3 and S6) comprises 2e/4c-bonded dimers of TCNE^•–^ radicals with C···C distances of only 2.8087(3) Å. Another anion–anion contact is present, between different dimers, which are offset approximately in the direction of one of the cyano-groups by ca. 1.58 Å. Therefore, interatomic distances in this contact are longer, with the closest being 3.2401(4) Å. In addition, there are eight symmetry-independent C–H···N hydrogen bonds (Table S3) between cations and anions and C–H···π interactions between methyl groups and aromatic rings of cations (Table S4). Four of these hydrogen bonds are long and do not satisfy commonly used criteria for hydrogen bonding^13^ but nevertheless exhibit a (3, −1) critical point (cp, Table S2). This discrepancy between the assignment of hydrogen bonding according to geometric and topological parameters was noted previously^33,34^ and is something rather common. One must remember when determining the presence of hydrogen bonds in a crystal structure that criteria based on electron density distribution are generally considered more reliable than those based solely on geometry. Electron density distribution provides direct insights into the nature of the bonding interactions, offering a clearer and more definitive identification of a hydrogen bond. In contrast, geometric criteria can sometimes be ambiguous or misleading as they rely on distances and angles that might not fully capture the nuances of the bonding interactions. Although the above-mentioned geometrical criteria are not fulfilled here, the full topological analysis of electron density, both from experiment and theory, indicates that all of the interactions fall within hydrogen bond criteria. Therefore, although geometric criteria can be useful for initial assessments, electron density distribution offers a more accurate and comprehensive approach for identifying hydrogen bonds.

**Figure 4:**
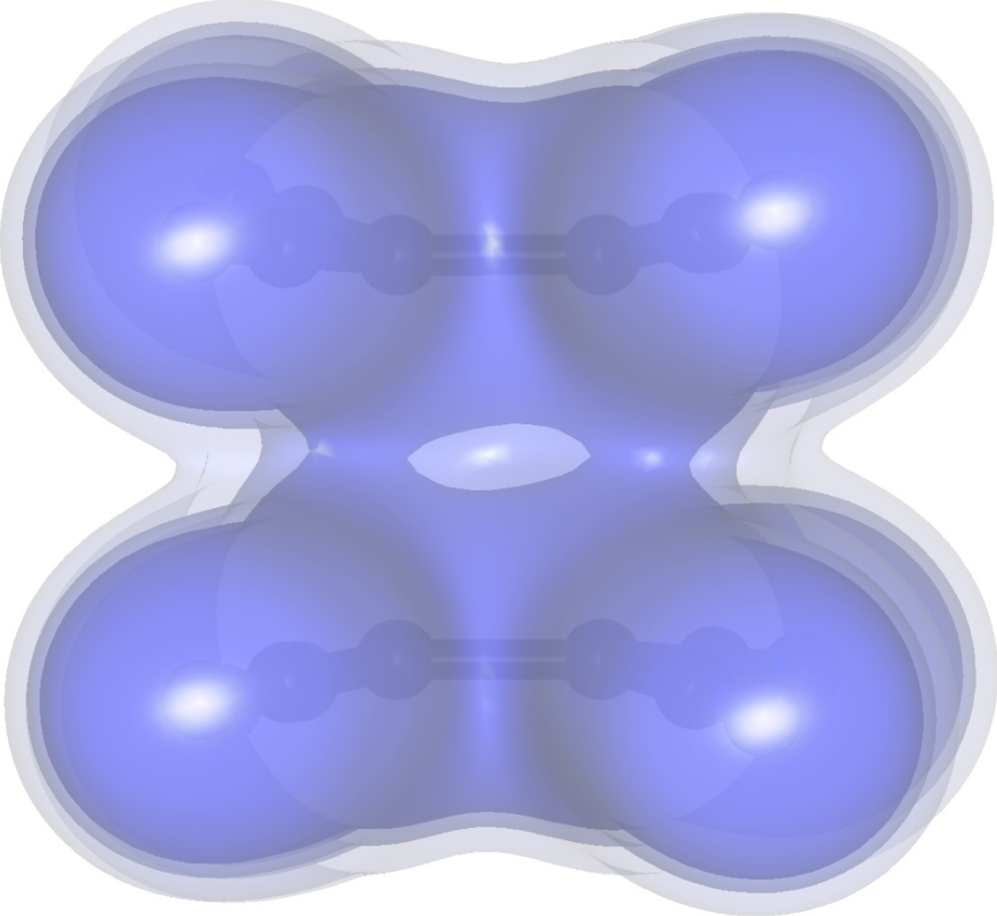
Experimentally determined electron density in a dimer of TNCE^•–^ radicals: isosurfaces of electron densities of 0.08, 0.12, and 0.17 e Å^–3^ are shown as light, medium, and dark shades, respectively.

**Figure 5:**
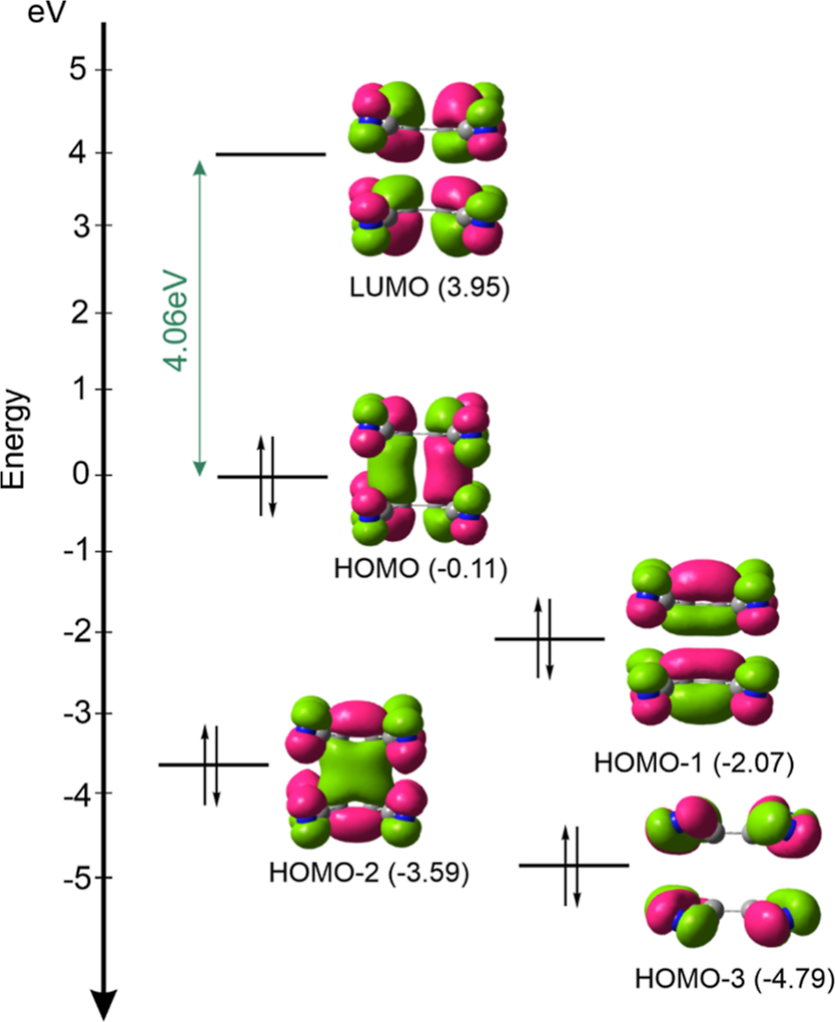
Selected HOMO-*n* and LUMO molecular orbitals of a dimer of interacting TCNE^•–^ radical anions in **1**. Value given in green refers to HOMO–LUMO gap. MO surfaces are drawn at isovalue of 0.025 au. DFT calculations were performed on nonoptimized dimer extracted from crystal structure.

The multipolar model revealed a considerable electron density between two central ethylene fragments C2≡C3 (Figure 4), which is in agreement with previously proposed models.^17−21^ Two inversion-related bond critical points (bcp’s) are found in a dimer of two TCNE^•–^ radicals (Figure 3), connecting by bond paths atoms C2···C3^*i*^ and C3···C2^*i*^ [symmetry operator (*i*) 1 – *x*, 1 – *y*, −*z*], again in accordance with the existence of a four-center two-electron π-bond. Experimentally determined maximum electron density in these bcp’s is 0.185 e Å^–3^, while the theoretical model yielded slightly smaller value of 0.118 e Å^–3^ (Table 2). These values are much higher compared to multicenter bonding in dimers of semiquinone radicals;^33−35^ however, the electron pair here is distributed over a much smaller area, and only two bcp’s are involved. Within a dimer, electron density is the lowest at a (3, +1) critical point, 0.156 e Å^–3^, indicating that the electron pair is indeed shared by four C atoms. The presence of 2e/4c bonding was confirmed by gas-phase quantum chemical computation. Frontier orbitals (Figure 5) are in agreement with the proposed model,^17^ and its ground state is a singlet (Table S5). A large HOMO–LUMO band gap of 4.06 eV indicates that **1** is an insulator.

**Figure 6:**
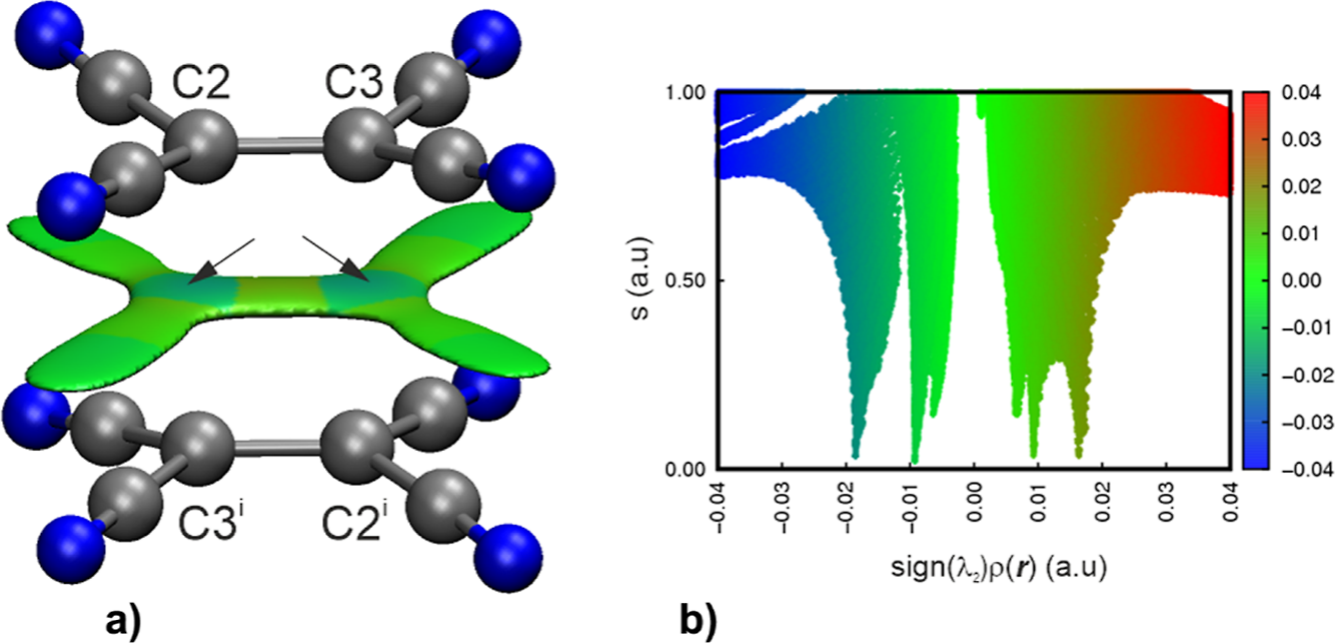
Reduced density gradient (RDG) isosurfaces (a) and fingerprint plots of the RDG against electron density multiplied by the sign of the second eigenvalue λ_2_ of the Hessian matrix (b), plotted for 2e/4c bonding in the TCNE^•–^ anion radicals truncated from the crystal structure of **1**. Gradient surfaces are plotted at 0.1 au level. Color scheme of the fingerprint plots and RDG isosurfaces: blue for attractive interactions, green for van der Waals, and red for repulsive interactions. Black arrows refer to bluish wide spike on fingerprint plots indicating inter-radical attractive interaction. Symmetry codes: (*i*) 1 – *x*, 1 – *y*, −*z*.

This interaction between TCNE^•–^ radical anions is nicely reflected in the NCI-index (noncovalent interactions analysis)^36,37^ as well as energy densities at the identified bcp’s. NCI combines electron density ρ(**r**) and its reduced density gradient (RDG)s(**r**) and features interactions in the shape of peaks in the low-gradient and low-density region. Multiplied by the second eigenvalue λ_2_ of the ED Hessian matrix, it allows us to determine whether an interaction has a stabilizing or destabilizing character. The multishaped RDG domain indicates multicenter inter-radical contacts between two moieties of TCNE^•–^ radical anions (Figure 6a), which is well reflected in fingerprints plots (Figure 6b) where most of the spikes are in the area of −0.01 < sign (λ_2_ρ)(***r***) < 0.01. Additional bluish spikes at around −0.02 (also marked with black arrows in Figure 6a) indicate a strong inter-radical interaction. Energy densities, in particular, the ratio of |*V*_BCP_(**r**)|/*G*_BCP_(**r**) (as previously mentioned in the context of hydrogen bonds), on the other hand offer valuable insights into whether an interaction is shared (e.g., covalent), closed-shell (e.g., hydrogen bond), or of intermediate character. If the ratio exceeds 2, the interaction is covalent. If it is below 1, it is a closed-shell interaction.^38^ A ratio between 1 and 2 indicates intermediate character. In our study, the ratio is approximately 1.18 based on the experimental data and 0.88 based on the theoretical data, suggesting rather intermediate to weak electrostatic character.

The longer contact between the dimers also involves two symmetry-equivalent (3, −1) cp’s, albeit with a much lower electron density, 0.068 e Å^–3^, between atoms N1 and C3^*ii*^ [symmetry operator (*ii*) 2 – *x*, 1 – *y*, −*z*]. The lowest electron density at the (3, +1) cp is 0.055 e Å^–3^. This is comparable to weaker multicenter bonding in stacks of equidistant semiquinone radicals^33^ and C–H···O hydrogen bonding.^39,40^ The electron density in weak stacking contacts between aromatics (and between dimers of semiquinone radicals) is typically lower than 0.040 e Å^–3^. However, since the electron pair in this case is spread over a smaller area than that in the semiquinone stacks and the HOMO orbital does not extend between the dimers (Figure S11), this interaction can be considered as nonbonding.

Since only a few charge-density studies deal with 2e/mc bonds in a rather disparate set of radicals, little is yet known from their comparison. However, a quite obvious general trend is that the maximum electron density is reduced as the radicals get larger (i.e., more centers involved in pancake bonding), and the distance between the radicals increases (Table 4). Therefore, maximum electron density in a TCNE^•–^ dimer is 2–3 times higher than that in dimers and trimers of semiquinone radicals;^33−35,41^ however, an outlier is a dimer of *N*,*N*,*N*′,*N*′-tetramethyl-*p*-phenylenediamine (TMPD) radical cations, whose considerably lower maximum electron density can be explained by a lack of prominent π-holes (which facilitate close contact of radicals), steric repulsion of methyl groups and a positive charge.^35^ Dithiadiazolyl radicals have the highest intermolecular electron density between its –SS– groups; for interatomic distances in the range 2.96–3.06 Å, the maximum electron densities are 0.143–0.166 e Å^–3^.^42,43^ However, due to different sizes and polarizabilities of C and S atoms, a direct comparison of these values to TCNE and the semiquinones is questionable.

**Table 4:** Comparison of Maximum Electron Density in Different Pancake Bonds

type of bond	no. of C centers	*d*/Å	ρ_max_/e Å^–3^	references
TCNE^•–^ dimer	4	2.864	0.185	this work
Cl_4_Q^•–^ dimer	12	2.864	0.095	(33)
DDQ^•–^ dimer	14	2.875	0.085	(34)
Cl_4_Q^•–^ trimer	18	2.839	0.077	(41)
TMPD^•+^ dimer	12	3.119	0.055	(35)
stack of equidistant Cl_4_Q^•–^		3.167	0.048	(33)

## Conclusions

Experimental and theoretical charge density of **1** as the prototype of the two-electron four-center bonding allowed us to establish a benchmark for future studies of two-electron multicenter π-bonding. It will be of use in experimental charge density and QTAIM studies on other systems with pancake-bonded radicals. The electron density between TCNE^•–^ radical anions is the highest intermolecular electron density expected in two-electron multicenter π-bonding, since it has the fewest centers, and therefore the electron pair is distributed over the smallest area.

Two symmetry-equivalent bcp’s were found between the C=C fragments of two contiguous TCNE^•–^ radical anions, and their experimental and theoretical maximum electron densities are 0.185 and 0.118 e Å^–3^. The electron density minimum within a dimer, a (3, +1) cp, has an experimental electron density of 0.156 e Å^–3^. Thus, the electron pair is indeed shared by four C atoms in two contiguous TCNE^•–^ radical anions.

The electron density in the nonbonding contact between two dimers of TCNE^•–^ radical anions is also much higher than between dimers of other radicals, which is due to a smaller contact area and relatively close C···C distances. Therefore, two symmetry-equivalent bcp’s are also found, with a maximum electron density of 0.068 e Å^–3^. Again, the relatively high electron density (for nonbonding contacts, it is usually <0.04 e Å^–3^)^1,2^ is due to a close contact between C and N atoms of neighboring dimers.

The comparison of the charge densities of the TCNE^•–^ radical anion and the neutral TCNE reveals that the majority of the negative charge in the radical anion is located in the central ethylene fragment; however, the cyano groups stabilize the anion by the inductive effect.

## Experimental Section

### Preparation

The glassware was dried at 120 °C overnight and transferred to an argon-filled glovebox (MBraun MB200B). TCNE (96%, Merck) was used without further purification. *N*-Methylpyridinium iodide was prepared, as described previously.^44^ Acetonitrile (≥99.9%, CHROMASOLV Gradient, for HPLC, Honeywell) was purified using the solvent purification system (Vigor VSPS-5) and stored over the 3 Å molecular sieves in the glovebox.

The reaction was carried out in an argon-filled glovebox (MBraun MB-200B), following a slightly modified literature procedure.^32^ TCNE (100 mg, 0.781 mmol) was added to a Schlenk flask and dissolved in dry acetonitrile (1 mL). A solution of *N*-methylpyridinium iodide (285 mg, 1.29 mmol, 1.65 equiv) in dry acetonitrile (1 mL) was added dropwise with vigorous stirring. Stirring was continued at room temperature for 30 min. Afterward, the reaction vessel was transferred out of the glovebox and connected to a vacuum line. The solution was concentrated in vacuo to approximately 0.5 mL and placed in a freezer at −18 °C. After a day, dark crystals of **1** precipitated out of the mixture. The reaction mixture was then cooled to −50 °C, and the solvent was removed in vacuo. Dark crystals of **1** were transferred to the low-temperature mounting apparatus, where the single crystal suitable for X-ray diffraction was selected under the flow of cold nitrogen stream (−50 °C).^45^

Single crystals of cubic TCNE were grown by slow evaporation of an ethyl acetate solution, as described previously.^25^

### Quantum Chemical Computations

The nature of intra- and intermolecular interactions has been studied by means of deformation density using periodic DFT calculations, performed with *CRYSTAL17* software.^46^ Atomic coordinates were utilized from final experimental multipolar refinement, with no further geometry optimization, and the compound was modeled on the PBE0/POB-TZVP theory level.^47^ A mesh of 8 × 8 × 8 *k*-points in reciprocal space was generated according to the Monkhorst–Pack method,^48^ and the condition for the self-consistent field convergence was set to 10^–10^ on the total energy difference between two subsequent cycles. Static structure factors were computed from the resultant wave functions up to the same resolution as that observed from the experiment and used in refinement with the XD2006 package. No thermal or positional parameters were refined in this model. Multipolar refinement on theoretical data was carried out up to the same level as the one used for the experimental charge density modeling to compare the obtained results with the experimental structure factors. Additionally, the electronic structure as well as properties of the two interacting TCNE^•–^ radical anions were evaluated using single-point DFT gas-phase calculations. For that purpose, unrestricted long-range corrected hybrid CAM-B3LYP density functional^49^ was used. Aug-cc-pVTZ basis set^50^ was employed to describe the radical anions with an appropriate set of diffuse functions. Atomic coordinates were taken from high-resolution X-ray diffraction experiments and were kept frozen during modeling. Although a dimer composed of two TCNE^•–^ radical anions should adopt a singlet ^1^A_g_ ground state,^17^ the system was also tested for triplet and quintet ground states. The obtained results confirmed singlet ground state behavior (see more details in the Supporting Information file in Computational Details). Gas-phase wave function was further used to perform quantitative NCI analysis by generating RDGs and calculating electron density-derived properties within the RDG regions. All NCI related calculations were carried out using *NCIPLOT4* software.^51^

### X-ray Diffraction and Multipolar Refinement

Single crystal of **1** was measured at 100.0(1) K on a Rigaku Oxford Diffraction XtaLAB Synergy-S, Dualflex, Eiger2 R CdTe 1 M diffractometer using a microfocused Ag Kα radiation to the maximum resolution of 0.45 Å. Single-crystal measurement for neutral TCNE was performed on a Rigaku Oxford Diffraction XtaLAB Synergy S diffractometer with a HyPix600 detector at 100.0(1) K using Mo Kα radiation to a maximum resolution of 0.45 Å. Data reduction and absorption correction were performed by *CrysAlis PRO* program package.^52^ The multiple integrated reflections were averaged for the corresponding space groups using *SORTAV*(53) adapted to the area detector data.

Independent-atom models were refined using *SHELXL*(54) with all non-hydrogen atoms refined anisotropically. Multipolar refinement was carried out vs all reflections *F*^2^ with program package MoPro;^55^ in the final refinement cycles, the data were cut off at *s* = 1.0 Å^–1^. O, N, and C were modeled as octupoles and hydrogens as dipoles; loose restraints were used for multipoles and kappas of chemically equivalent atoms. Local symmetry restraints were applied to the multipolar parameters. Kappas of the hydrogen atoms were restrained to 1.15(1). Aromatic C–H bond lengths were restrained to 1.077(2) Å, and methyl C–H bond lengths were restrained to 1.083(2) Å. Geometry and charge-density calculations were performed by *MoPro*;^55^ molecular graphics were prepared using *MoProViewer*,^56^*ORTEP-3*,^57^ and Mercury.^58^ Crystallographic and refinement data are listed in Table 5.

**Table 5:** Crystallographic, Data Collection, and Charge-Density Refinement Details

compound	**1**	neutral TCNE
empirical formula	C_12_H_8_N_5_	C_6_N_4_
formula wt/g mol^–1^	222.23	126.10
crystal dimensions/mm	0.34 × 0.28 × 0.21	0.28 × 0.25 × 0.14
space group	*P*2_1_/*n*	*Im*3̅
*a*/Å	6.75930(1)	9.6285(3)
*b*/Å	10.0841(1)	9.6285(3)
*c*/Å	16.5374(2)	9.6285(3)
α/°	90	90
β/°	95.332(1)	90
γ/°	90	90
*Z*	4	4
*V*/Å^3^	1122.34(2)	892.65(4)
*D*_calc_/g cm^–3^	1.316	1.430
μ/mm^–1^	0.054	0.083
Θ range/°	2.49–38.55	2.99–53.96
*T*/K	100.0(1)	100.0(1)
radiation wavelength	0.56087 (Ag Kα)	0.71073 (Mo*K*α)
diffractometer type	Synergy S	Synergy S
range of *h*, *k*, *l*	–15 < *h* < 15	–21 < *h* < 21
	–22 < *k* < 22	–21 < *k* < 21
	–36 < *l* < 36	–21 < *l* < 21
reflections collected	155,518	52,896
independent reflections	9170	1012
reflections with I ≥ 2σ	8026	939
absorption correction	analytical	analytical
*T*_min_, *T*_max_	0.982, 0.989	0.469, 1.000
*R*_int_	0.0854	0.0407
spherical refinement		
weighting scheme	*w* = 1/[σ^2^(*F*_o_^2^) + (0.0852*P*)^2^ + 0.0416*P*] where *P* = (*F*_o_^2^ + 2*F*_c_^2^)/3	*w* = 1/[σ^2^(*F*_o_^2^) + (0.082*P*)^2^ + 0.0787*P*] where *P* = (*F*_o_^2^ + 2*F*_c_^2^)/3
*R* (*F*)	0.0424	0.0306
*R*_w_ (*F*^2^)	0.1469	0.1576
goodness of fit	1.054	1.439
H atom treatment	constrained isotropic	
no. of parameters	155	17
no. of restraints	0	0
Δρ_max_, Δρ_min_, Δρ_rms_ (e Å^–3^)	0.547; −0.274; 0.053	0.872; −0188; 0.087
multipolar refinement		
weighting scheme	*w* = 1/[σ^2^(*F*_0_^2^)]	*w* = 1/[σ^2^(*F*_0_^2^)]
*R* (*F*)	0.0240	0.0103
*R*_w_ (*F*^2^)	0.0615	0.0305
goodness of fit	1.180	1.063
H atom treatment	constrained isotropic	
no. of parameters	524	82
no. of restraints	330	0
Δρ_max_, Δρ_min_, Δρ_rms_ (e Å^–3^)	0.471; −0.253; 0.025	0.126; −0.147; 0.016

Topological bond orders were calculated using following formula^59^
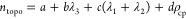


Coefficients *a*, *b*, *c*, and *d* were taken from the literature: for C–C bonds, *a* = −0.522, *b* = −1.695, *c* = 0.00, and *d* = 8.473;^60^ for C–N bonds, *a* = −0.284, *b* = 0.331, *c* = 0.559, and *d* = 6.569;^60^ and for C–H bonds, *a* = −0.153, *b* = 0.481, *c* = 0.983, and *d* = 8.087.^61^

## Supporting Information Available

Details on charge density of **1** and neutral TCNE; details on crystal packing of **1**; and additional details on quantum chemical computations (PDF)

## Abbreviations

DFT: density functional theory
TCNE: tetracyanoethylene
TCNE^•–^: tetracyanoethylene radical anion
HOMO: highest occupied molecular orbital
LUMO: lowest unoccupied molecular orbital
2e/4c: two-electron four-center bonding
RDG: reduced density gradient
QTAIM: quantum theory of atoms in molecules
ED: electron density
cp: critical point
bcp: bonding critical point
Cl4Q^•−^: tetrachlorosemiquinone
DDQ^•−^: 5,6-Dichloro-2,3-dicyanosemiquinone
