# Supplementary material for: Charge Density Study of Two-Electron Four-Center Bonding in a Dimer of Tetracyanoethylene Radical Anions as a Benchmark for Two-Electron Multicenter Bonding
Source: Cryst Growth Des. 2024 Jul 22;24(15):6187–95. doi: 10.1021/acs.cgd.4c00342 (PMC11311126; doi:10.1021/acs.cgd.4c00342)
Supplement: Supplementary file 1 — cg4c00342_si_001.pdf [file cg4c00342_si_001.pdf]

Charge density study of two-electron four-center bonding in a dimer of tetracyanoethylene radical anions as a benchmark for two-electron multicenter bonding

Supporting Information

Miha Virant,[a]* Petar Štrbac,[b] Anna Krawczuk,[c] Valentina Milašinović,[a,b] Petra Stanić,[b] Matic Lozinšek,[a] Krešimir Molčanov*[b]

[a] Jožef Stefan Institute, Jamova cesta 39, SI-1000, Ljubljana, Slovenia

[b] Ruđer Bošković Institute, Bijenička 54, HR-10000 Zagreb, Croatia

[c] University of Göttingen, Tammanstrasse 4, D-37077 Gottingen, Germany

e-mail: kmolcano@irb.hr, miha.virant@ijs.si

S1 Details on charge density of **1**

S2 Details on crystal packing of **1**

S3 Details on charge density of neutral TCNE

S4 Additional details on quantum chemical computations

S5 List of structures with TCNE radical anions from the Cambridge Structural Database

S1 Details on charge density of **1**

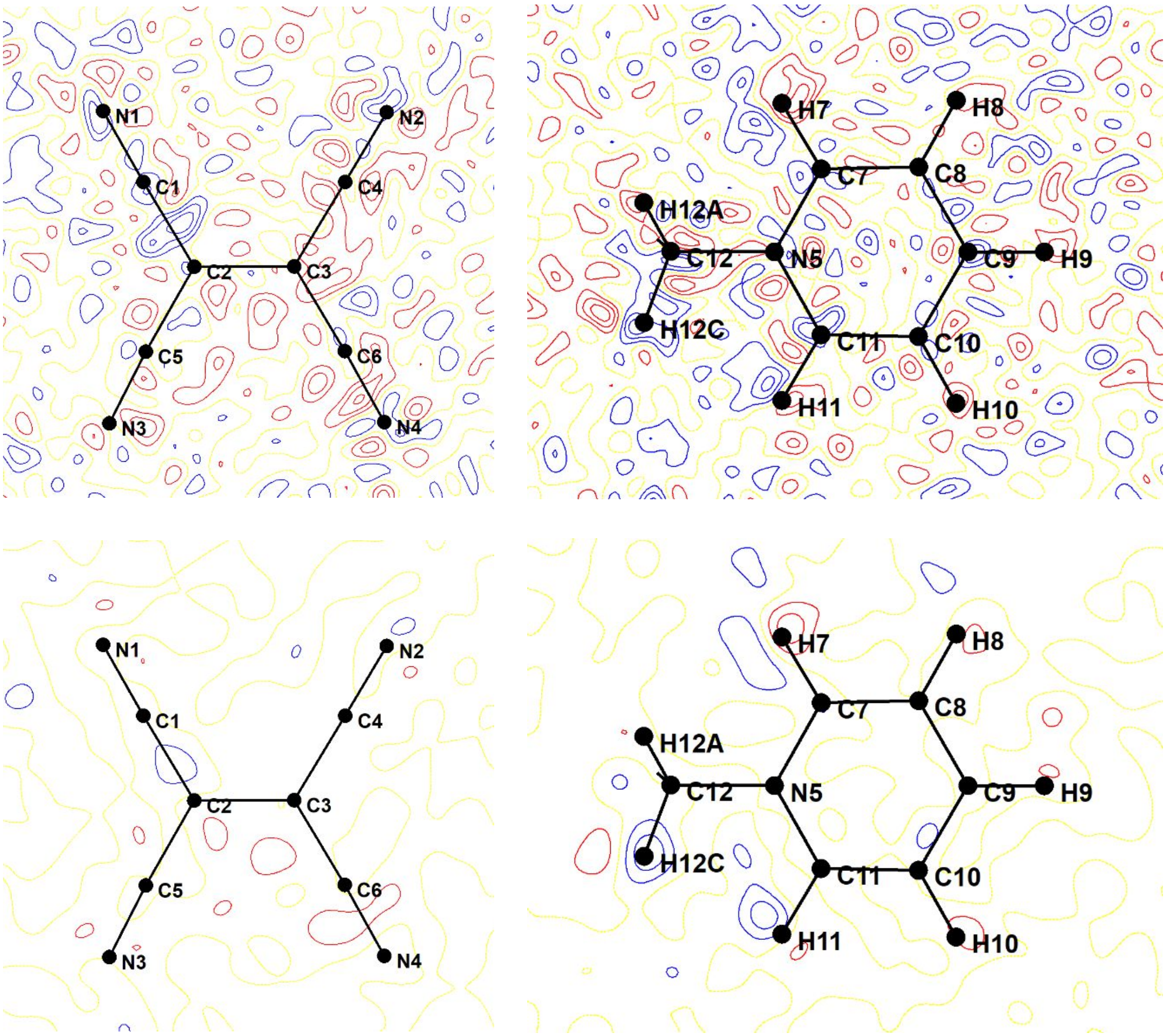

Figure S1 Residual electron density of the multipole refinement at the tetracyanoethylene radical anion (left) and *N*-methylpyridinium cation (right) planes with all reflections used (top row) and only low-angle reflections ($s < 0.7$ Å^{-1}) used in the refinement (bottom row). Positive density is shown in blue and negative in red; yellow dotted lines represent zero density. Contours are drawn at the 0.05 e Å^{-1} level.

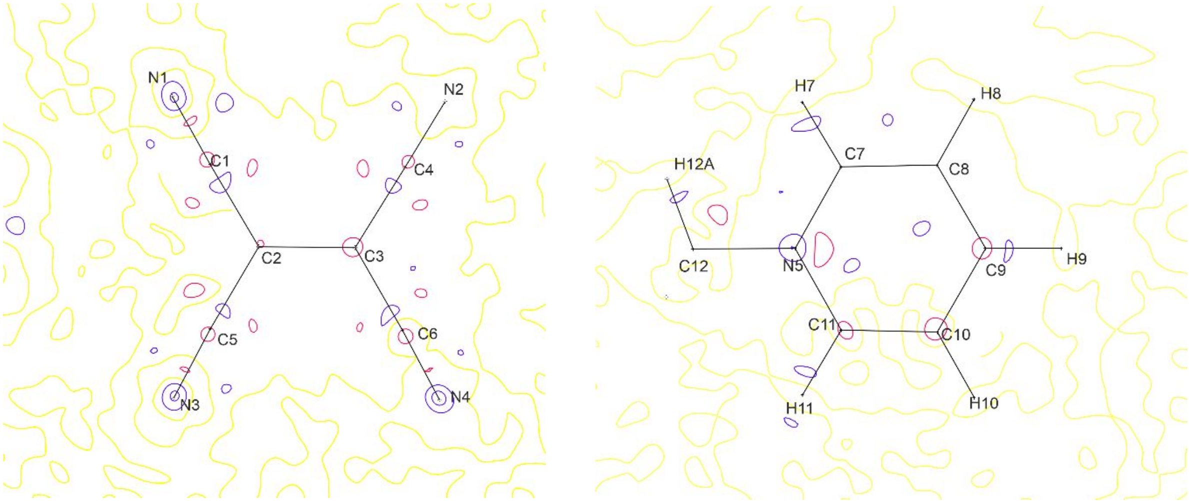

Figure S2 Residual electron density of the multipole refinement performed on theoretically obtained structure factors. Positive density is shown in blue and negative in red; yellow lines represent zero density. Contours are drawn at the 0.05 e Å^{-1} level.

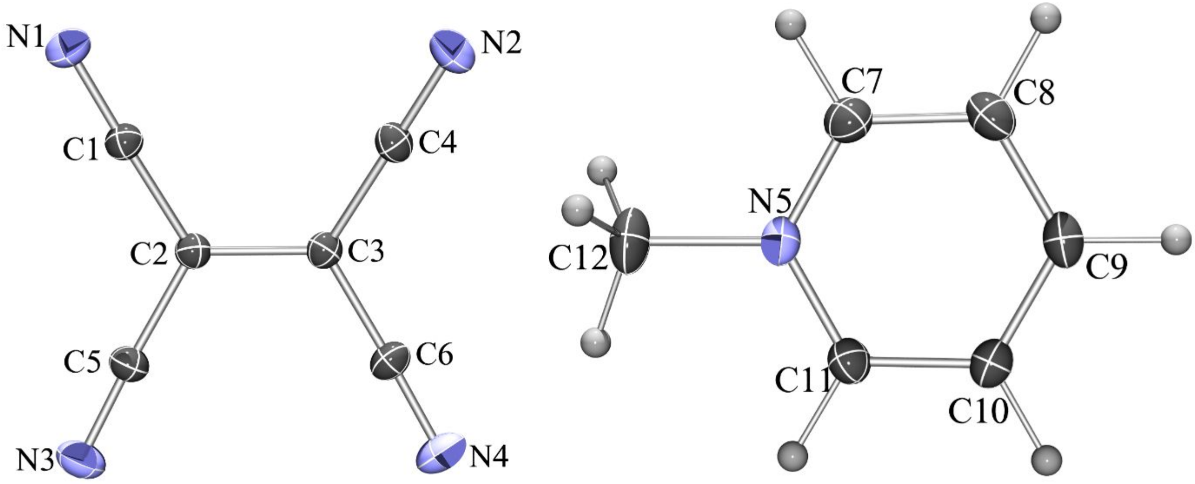

Figure S3 ORTEP-3 drawings of $TCNE^{\bullet-}$ radical anion (left) and *N*-methylpyridinium cation (right) with atom numbering scheme. Displacement ellipsoids are drawn at the 50% probability and hydrogen atoms are shown as spheres of arbitrary radii.

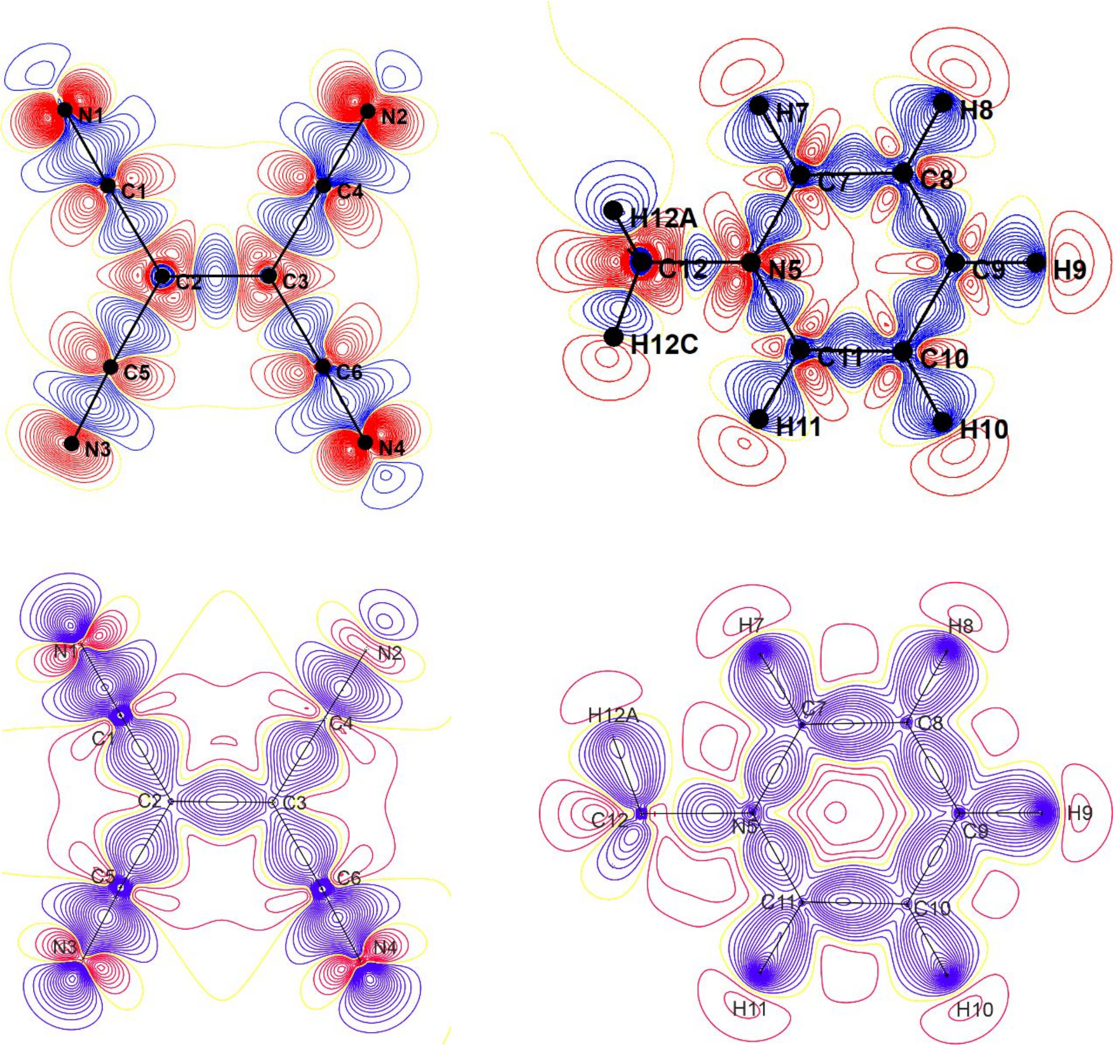

Figure S4 Experimental (top row) and theoretical (bottom row) deformation density maps of TCNE$^{\bullet-}$ radical anion (left), and *N*-methylpyridinium cation (right). Contours are drawn at the 0.05 e Å^{-3} electron density level; positive density is blue, negative is red and zero contour is drawn as a yellow dotted line.

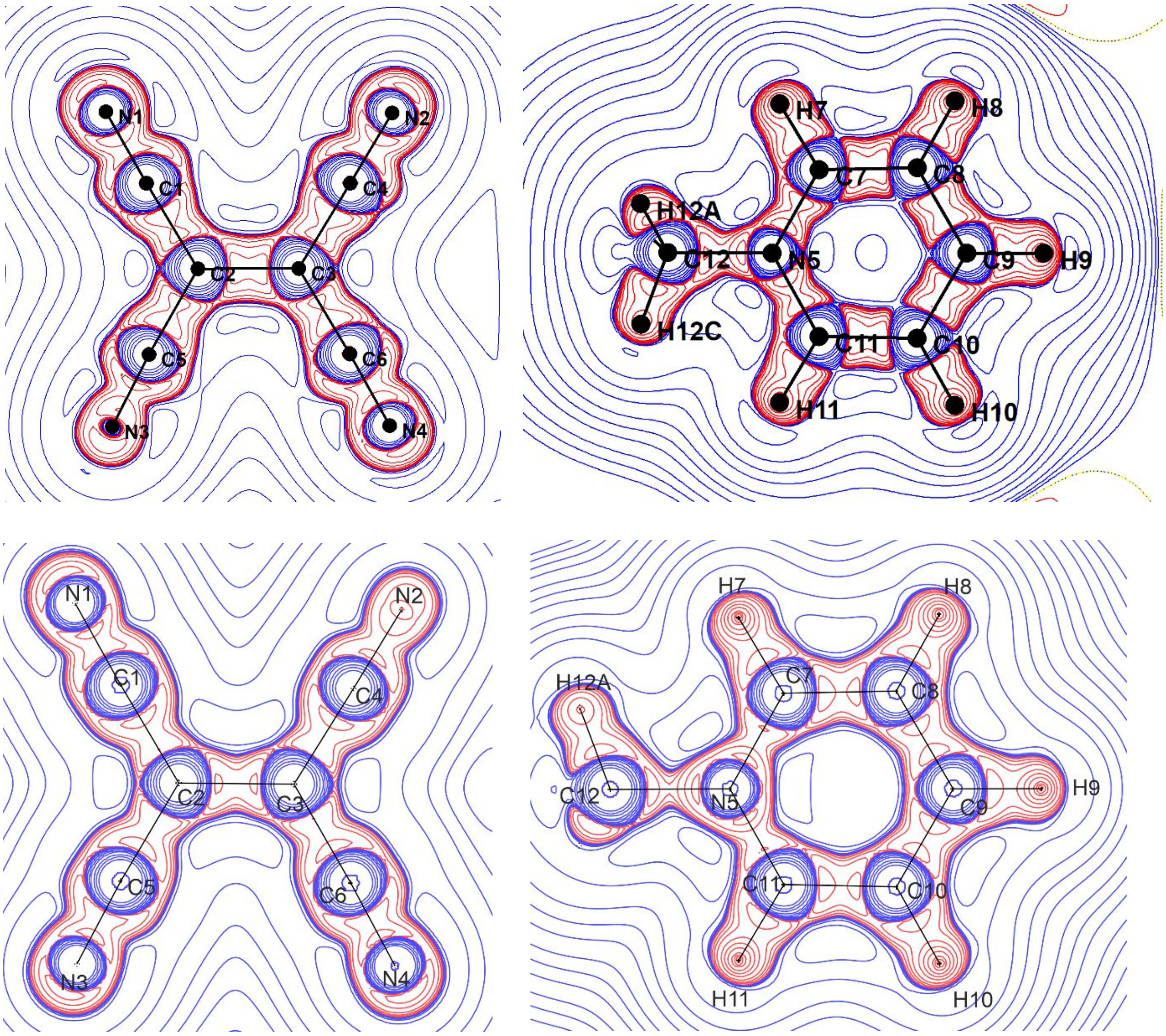

Figure S5 Experimental (top row) and theoretical (bottom row) Laplacians of electron density for $TCNE^{\bullet-}$ radical anion (left) and *N*-methylpyridinium cation (right). Contours: 2, 4, 8 × 10^n e/$Å^{-5}$, $n = -3…2$.

Table S1 Topology of electron density in the *N*-methylpyridinium cation, derived from experimental (regular font) and theoretical (italic) electron-density after multipole refinement.

Bond	Length (Å)	Electron Density (e Å^{-3}) ρ_{cp}	Laplacian (e Å^{-5})	Ellipticity	Bond order n_{topo}
N5−C7	1.3473(4)	2.043	−13.9	0.08	1.22
		2.153	*−19.8*	*0.08*	
N5−C11	1.3457(4)	2.083	−14.8	0.01	1.23
		2.153	*−19.9*	*0.10*	
N5−C12	1.4678(4)	1.751	−19.5	0.06	0.88
		1.619	*−7.9*	*0.04*	
C7−C8	1.3809(4)	2.102	−21.0	0.25	1.59
		2.104	*−17.3*	*0.14*	
C8−C9	1.3879(5)	2.060	−19.9	0.19	1.46
		2.073	*−17.3*	*0.14*	
C9−C10	1.3870(4)	2.046	−20.1	0.19	1.45
		2.087	*−17.5*	*0.14*	
C10−C11	1.3800(4)	2.103	−21.3	0.28	1.59
		2.117	*−18.0*	*0.19*	
C7−H7	1.083(4)	1.734	−18.3	0.06	0.90
		1.802	−15.7	*0.04*	
C8−H8	1.083(3)	1.738	−17.1	0.04	0.95
		1.783	−15.5	*0.02*	
C9−H9	1.084(4)	1.751	−19.2	0.02	0.93
		1.806	*−15.9*	*0.01*	
C10−H10	1.083(4)	1.763	−17.5	0.06	0.90
		1.788	*−15.6*	*0.02*	
C11−H11	1.083(5)	1.695	−18.0	0.05	0.87
		1.803	*−15.9*	*0.05*	
C12−H12A	1.095(2)	1.461	−14.2	0.11	0.79
		1.746	*−14.2*	*0.04*	
C12−H12B	1.095(1)	1.629	−14.0	0.14	0.99
		1.751	*−14.3*	*0.04*	
C12−H12C	1.095(1)	1.735	−16.8	0.14	0.85
		1.751	*−14.1*	*0.04*	

S2 Details on crystal packing of **1**

Table S2 Experimentally (first row, regular font) and theoretically (second row, italic font) determined intermolecular critical points in **I**. Hydrogen bonds which have a BCP, but geometry is unfavourable (in Table S3) are printed in italic in the first column.

A···B	Electron Density (e Å^{-3}) ρ_{cp}	Laplacian (e Å^{-5})	type	Symm. operation on *B*	Potential energy density (e Å^{-5})	Kinetic energy density (e Å^{-5})
2e/4c bonding						
C2···C3	0.185	1.25	(3,−1)	1−*x*, 1−*y*, −*z*	*−0.13*	*0.11*
	0.118	*1.15*			*−0.07*	*0.08*
N1···C3	0.068	0.70	(3,−1)	2−*x*, 1−*y*, −*z*	*−0.03*	*0.04*
	0.039	*0.52*			*−0.02*	*0.02*
N1···N1	0.055	0.45	(3,+1)	2−*x*, 1−*y*, −*z*	0.00	0.00
	not found					
C3···C2	0.111	1.22	(3,+1)	1−*x*, 1−*y*, −*z*		
	not found					
hydrogen bonding						
H12B···N1	0.047	0.57	(3,−1)	1−*x*, 1−*y*, −*z*	−0.06	0.05
	0.037	*1.57*			*−0.02*	*0.03*
H12C···N2	0.040	0.42	(3,−1)	−1/2+*x*, 1/2−*y*, 1/2+*z*	−0.02	0.01
	0.024	*0.36*			*−0.01*	*0.02*
H9···N2	0.064	0.85	(3,−1)	1−*x*, −*y*, −*z*	−0.02	0.01
	0.055	*0.85*			*−0.02*	*0.04*
H8···N2	0.047	0.60	(3,−1)	−1+*x*, *y*, *z*	−0.02	0.01
	0.039	*0.60*			*−0.02*	*0.03*
H8···N3	0.045	0.47	(3,−1)	−*x*, −*y*, −*z*	−0.01	0.00
	not found					
H11···N3	0.028	0.47	(3,−1)	3/2−*x*, −1/2+*y*, 1/2−*z*	−0.02	0.01
	0.016	*0.34*			*−0.01*	*0.02*
H12B···N4	0.044	0.51	(3,−1)	*x*, *y*, *z*	−0.02	0.01
	0.031	*0.45*			*−0.02*	*0.02*
H10···N4	0.060	0.74	(3,−1)	1−*x*, 1−*y*, −*z*	−0.02	0.02

	not found					
C−H···π						
H12B···C9	0.067	0.66	(3,−1)	1/2−*x*, 1/2+*y*, 1/2−*z*	−0.02	0.01
	0.036	*0.45*			*−0.02*	*0.03*
other						
N1···C7	0.064	0.67	(3,−1)	1–*x*, 1–*y*, –*z*		
	0.041	*0.52*				
N3···C8	0.045	0.47	(3,−1)	1–*x*, 1–*y*, –*z*		
	0.026	*0.33*				
N2···C10	0.042	0.45	(3,−1)	1–*x*, 1–*y*, –*z*		
	not found					
N4···C11	0.027	0.35	(3,−1)	*x*, *y*, *z*		
	0.028	0.37				
N4···C12	0.032	0.47	(3,-1)	*x*, *y*, *z*		
	not found					
N1···C11	0.054	0.61	(3,-1)	1/2+*x*, 1/2–*y*, –1/2+*z*		
	0.028	*0.43*				
N4···C7	0.039	0.43	(3,-1)	1/2–*x*, 1/2+*y*, 1/2–*z*		
	not found					
H9···H9	0.036	0.69	(3,-1)	1–*x*, –*y*, –*z*		
	0.030	*0.64*				

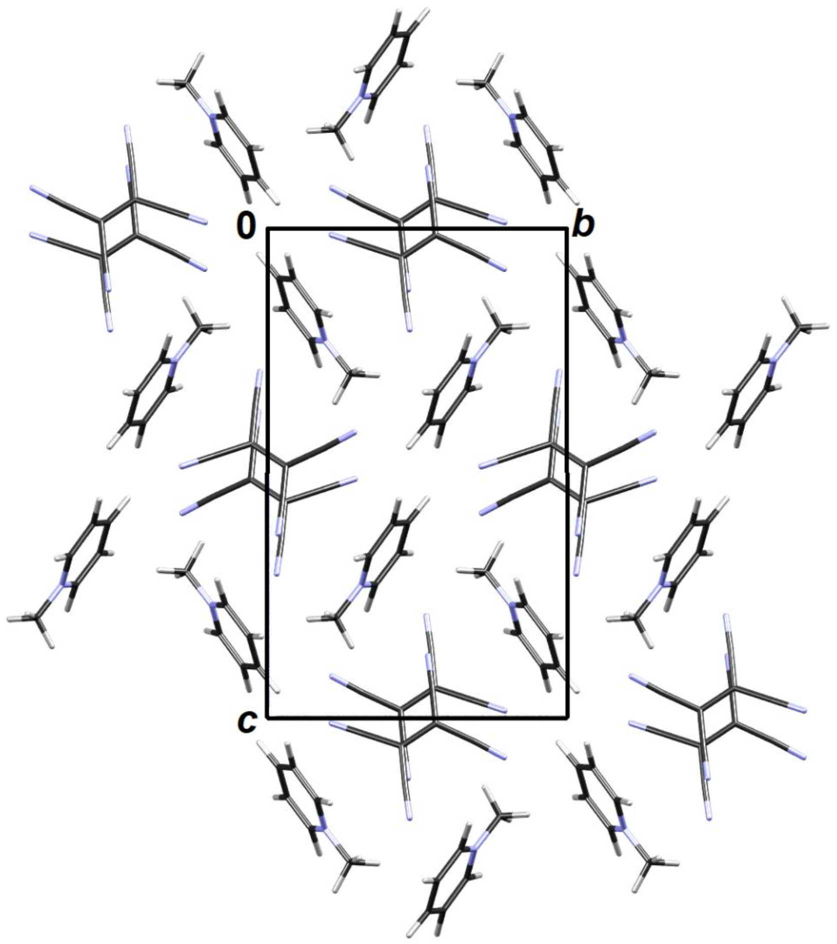

Figure S6 Crystal packing of **1** viewed in the [100] direction.

Table S3 Geometric parameters of hydrogen bonding (Å, °). Hydrogen bonds which have unfavourable geometry, but bcp is found (in Table S2) are printed in italic.

	D−HÅ	H···*A*/Å	*D*···*A*/Å	*D*–H···*A*/°	Symm. op. on *A*
C8−H8···N3	1.083(3)	2.693(5)	3.4032(3)	117.3(2)	−*x*, −*y*, −*z*
C9−H9···N2	1.084(4)	2.499(5)	3.2654(5)	126.8(3)	1−*x*, −*y*, −*z*
C10−H10···O4	1.083(4)	2.577(5)	3.2910(5)	122.8(3)	3/2−*x*, −1/2+*y*, 1/2−*z*
C12−H12B···N1	1.095(6)	2.682(2)	3.4721(3)	128.5(9)	1−*x*, 1−*y*, −*z*
C8−H8···N2	1.083(3)	2.652(4)	3.4029(5)	126.0(3)	−1+*x*, *y*, *z*
C11−H11···N3	1.083(5)	2.577(5)	3.846(6)	122.8(3)	3/2−*x*, −1/2+*y*, 1/2−*z*
C12−H12B···N4	1.0950(6)	2.8825(8)	3.4401(5)	111.61(3)	*x*, *y*, *z*
C12−H12C···N2	1.095(2)	2.9060(10)	3.554(6)	118.09(12)	−1/2+*x*, 1/2−*y*, 1/2+*z*

Table S4 Geometric parameters of C−H···π interactions (Å, °).

	D−H/Å	H···Cg/Å	*γ*/°	*D*–H···Cg/°	Symm. op. on *A*

C12−−H12B···N5→C11	1.095(6)	2.6447(6)	9.42	139.5(1)	1/2−*x*, 1/2+*y*, 1/2−*z*

S3 Details on charge density of neutral TCNE

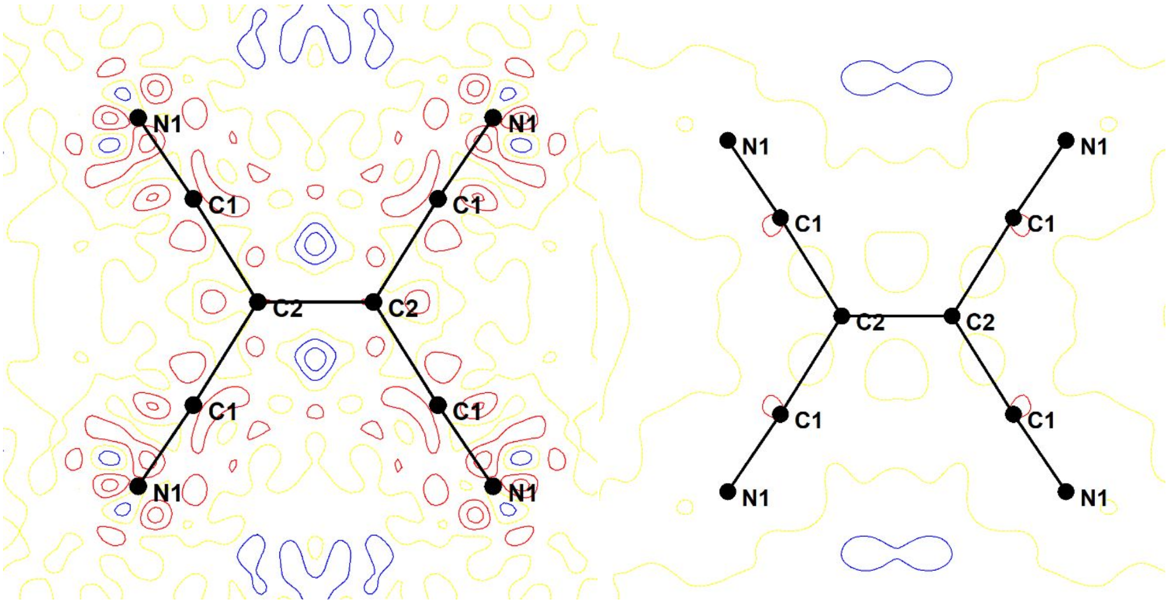

Figure S7 Residual electron density in the mean plane of the neutral tetracyanoethylene with all reflections used (left) and only low-angle reflections ($s < 0.7$ Å^{-1}) used (right). Positive density is shown in blue and negative in red; yellow dotted lines represent zero density. Contours are drawn at the 0.05 e Å^{-1} level.

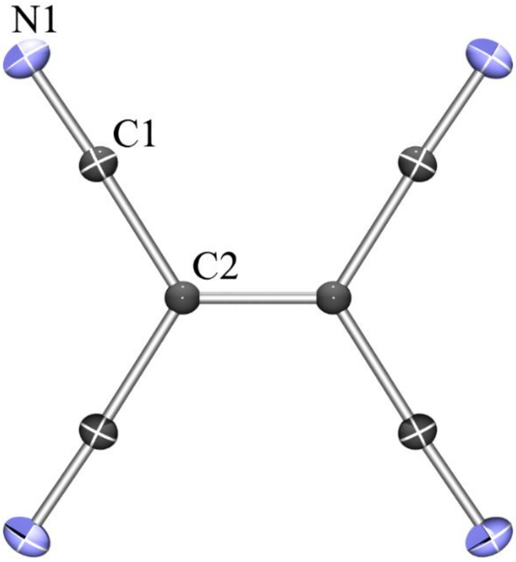

Figure S8 ORTEP-3 drawing of a neutral TCNE with atom numbering scheme. Displacement ellipsoids are drawn at the 50% probability level and hydrogen atoms are shown as spheres of arbitrary radii.

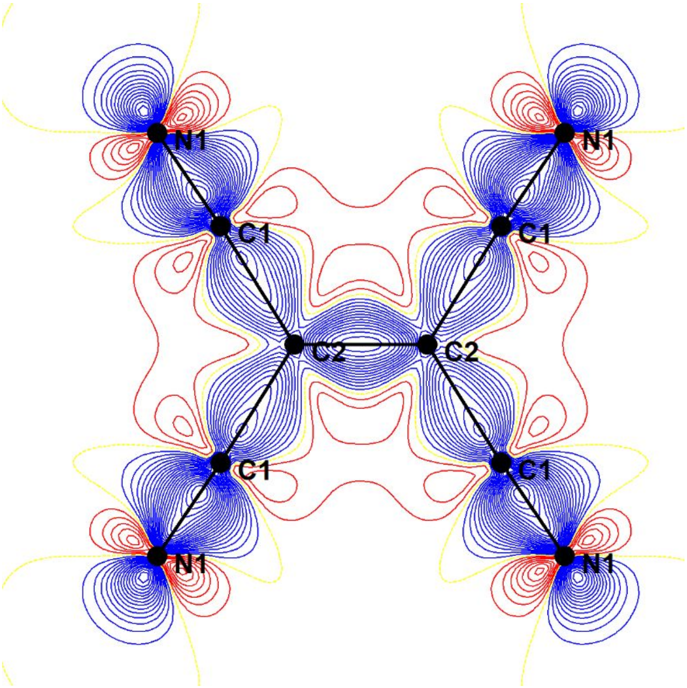

Figure S9 Deformation density map of neutral tetracyanoethylene. Contours are drawn at the 0.05 e Å^{-3} electron density level; positive density is blue, negative is red and zero contour is drawn as a yellow dotted line.

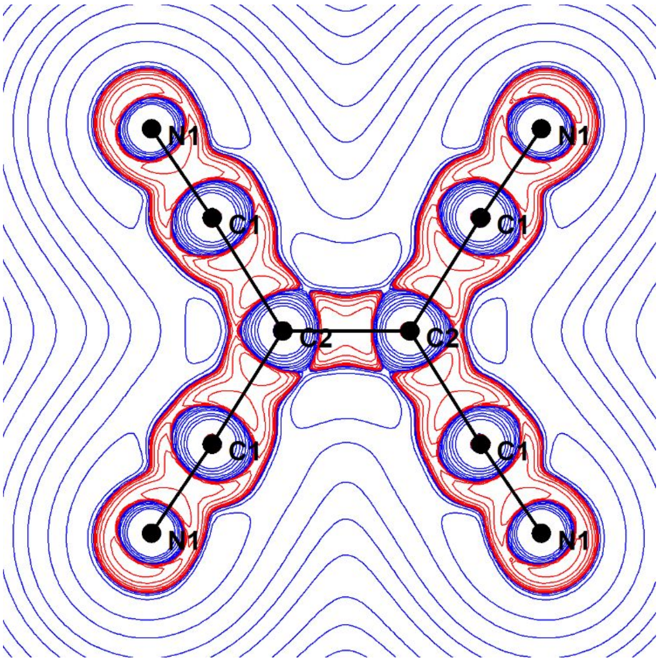

Figure S10 Laplacian of electron density for neutral tetracyanoethylene. Contours: 2, 4, 8 × 10^n e/Å^{-5}, $n = -3\ldots2$.

S4 Additional details on quantum chemical computations

To ensure that the interacting dimers are in the singlet state, a series of calculations with different electronic state, i.e. singlet, triplet and quintet was performed. Obtained energies (Table S5) indicate that indeed the system is in a singlet state, which is in agreement with previous findings [17].

Table S5 Energy calculations assuming different electronic state for the dimer build of two TCNE$^{\bullet-}$ radical anions

Electronic state	Charge / multiplicity	Energy (Hartree)
singlet	−2 / 0	−895.1000
triplet	−2 / 2	−895.0814
quintet	−2 / 4	−894.8994

In addition to calculations of dimer, a trimer composed of a dimer and a neighboring TCNE$^{\bullet-}$ radical anion was also computed. The resulting atomic charges (see Table S6) as well as molecular orbital behavior confirmed that binding interaction happens only between two of the three TCNE$^{\bullet-}$ radical anions, the ones that were taken for other dimer-related calculations.

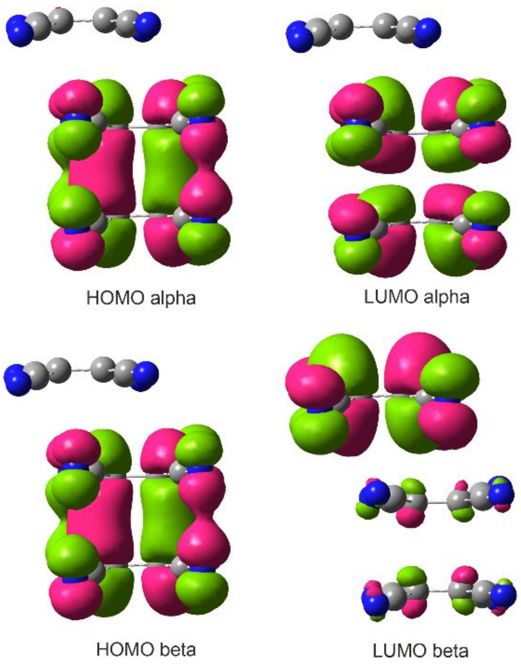

Figure S11 HOMO and LUMO molecular orbitals of a TCNE$^{\bullet-}$ radical anions trimer taken from the crystal structure of **1**. MO surfaces are drawn at the isovalue of 0.015 au. Alpha refers to positive spin (up), beta to negative spin (down).

Table S6 Theoretical Bader and Mulliken atomic charges calculated for TCNE$^{\bullet-}$ radical anions involved in stacking interactions. Calculations were done in gas-phase for both dimer or trimer case.

	**dimer**		**trimer***	
	**Bader charges**	**Mulliken charges**	**Bader charges**	**Mulliken charges**
N1	−1.208	−0.933	−1.165	−1.154
N2	−1.229	−0.906	−1.188	−1.159
N3	−1.204	−0.894	−1.188	−0.999
N4	−1.204	−0.900	−1.201	−1.032
C1	0.870	0.451	0.862	0.758
C2	0.178	0.426	0.197	0.253
C3	0.178	0.497	0.204	0.417
C4	0.887	0.460	0.870	0.701
C5	0.879	0.425	0.877	0.700
C6	0.868	0.375	0.877	0.494
**SUM**	**−0.985**	**−0.999**	**−1.021**	**−0.855**

* atomic charges are listed for the radical anion that is in between two other TCNE$^{\bullet-}$ radical anions

NCIPLOT4 integration data for the interacting dimer:

```
----------------------------------------------------------------------
                INTEGRATION DATA
----------------------------------------------------------------------
Integration over the volumes of rho^n
----------------------------------------------------------------------
n=1.0        :     4.96409757
n=1.5        :     0.93038354
n=2.0        :     0.18148703
n=2.5        :     0.03636875
n=3.0        :     0.00743292
n=4/3        :     1.61891121
n=5/3        :     0.53879791
Volume       :   177.35131392
rho-sum_i rho_i :     0.00179720
```

```
----------------------------------------------------------------------
Integration  over the areas of rho^n
----------------------------------------------------------------------
n=1.0        :    26.53766520
n=1.5        :     5.35433474
n=2.0        :     1.10391149
n=2.5        :     0.23080645
n=3.0        :     0.04876146
n=4/3        :     9.10195039
n=5/3        :     3.15710180
Area         :   760.78864156
rho-sum_i rho_i :     0.01092959
```

```
---------------------------------------------------------------------
Integration over the volumes of sign(lambda2)(rho)^n
---------------------------------------------------------------------
n=1.0        :     0.62246600
```

n=1.5	:	0.07818878
n=2.0	:	0.00893034
n=2.5	:	0.00071727
n=3.0	:	-0.00004131
n=4/3	:	0.15716255
n=5/3	:	0.03868805

Gaussian geometry input:

Dimer:

N(Fragment=1)	6.008733	5.380201	-2.052239
N(Fragment=1)	5.221012	2.137240	0.214659
N(Fragment=1)	4.699921	7.937347	1.361495
N(Fragment=1)	3.646785	4.761963	3.400454
C(Fragment=1)	5.477917	5.514941	-1.030069
C(Fragment=1)	4.825652	5.634732	0.220633
C(Fragment=1)	4.482019	4.480781	0.981295
C(Fragment=1)	4.869998	3.183001	0.558690
C(Fragment=1)	4.754404	6.917814	0.815327
C(Fragment=1)	4.024155	4.610235	2.313318
N(Fragment=2)	0.760867	4.707399	2.052239
N(Fragment=2)	1.548588	7.950360	-0.214659
N(Fragment=2)	2.069679	2.150253	-1.361495
N(Fragment=2)	3.122815	5.325637	-3.400454
C(Fragment=2)	1.291683	4.572659	1.030069
C(Fragment=2)	1.943948	4.452868	-0.220633
C(Fragment=2)	2.287581	5.606819	-0.981295
C(Fragment=2)	1.899602	6.904599	-0.558690
C(Fragment=2)	2.015196	3.169786	-0.815327
C(Fragment=2)	2.745445	5.477365	-2.313318

Trimer:

N(Fragment=1)	5.992638	5.382681	-2.060206
N(Fragment=1)	5.207825	2.130569	0.209446
N(Fragment=1)	4.692880	7.939010	1.356127
N(Fragment=1)	3.641967	4.754552	3.398220
C(Fragment=1)	5.468146	5.511364	-1.029938
C(Fragment=1)	4.820025	5.632373	0.218337
C(Fragment=1)	4.479545	4.478349	0.979652
C(Fragment=1)	4.865881	3.187181	0.555525
C(Fragment=1)	4.752444	6.911138	0.814252
C(Fragment=1)	4.026275	4.609745	2.309976
N(Fragment=2)	0.766662	4.701419	2.060206
N(Fragment=2)	1.551475	7.953531	-0.209446
N(Fragment=2)	2.066420	2.145090	-1.356127
N(Fragment=2)	3.117333	5.329548	-3.398220
C(Fragment=2)	1.291154	4.572736	1.029938
C(Fragment=2)	1.939275	4.451727	-0.218337
C(Fragment=2)	2.279755	5.605751	-0.979652
C(Fragment=2)	1.893419	6.896919	-0.555525
C(Fragment=2)	2.006856	3.172962	-0.814252
C(Fragment=2)	2.733025	5.474355	-2.309976
N(Fragment=3)	7.525962	4.701419	2.060206
N(Fragment=3)	8.310775	7.953531	-0.209446
N(Fragment=3)	8.825720	2.145090	-1.356127
N(Fragment=3)	9.876633	5.329548	-3.398220
C(Fragment=3)	8.050454	4.572736	1.029938
C(Fragment=3)	8.698575	4.451727	-0.218337
C(Fragment=3)	9.039055	5.605751	-0.979652
C(Fragment=3)	8.652719	6.896919	-0.555525
C(Fragment=3)	8.766156	3.172962	-0.814252
C(Fragment=3)	9.492325	5.474355	-2.309976

S5 List of structures with TCNE radical anions from the Cambridge Structural Database

Table S7 A list of structures with TCNE radical anions deposited in the Cambridge Structural Database (CSD). Structures with disordered TCNE moiety are omitted. Different types of interaction are colour-coded: σ-dimers are green, 2e/4c-bonded dimers are yellow, weakly interacting pairs of radicals are light blue, those forming π-complex with a metal are red, stacked charge-transfer system with tetrathiafulvalene is light gray and isolated TCNE's (forming no apparent interactions with other TCNE moieties) are brown. The geometric parameters *r* and *d* represent the closest distance between two C atoms of contiguous TCNE moieties and an angle between C=C axes, respectiely.

CSD refcode	*r* / Å	*d* [°]	RMSD plane of TCNE / Å	avg C-N / Å	sd C-N / Å	C=C / Å	avg C-C / Å	sd C-C / Å
**DIVCAN-pt1**	1.60	0	0.272	1.140	0.009	1.524	1.434	0.047
**NUHGOM**	1.63	0	0.292	1.139	0.016	1.508	1.442	0.052
**DIVCAN-pt2**	1.66	0	0.287	1.118	0.022	1.610	1.446	0.074
**PORVAU01**	2.80	0	0.116	1.154	0.004	1.423	1.410	0.004
**AGAMUR-pt1**	2.81	4	0.093	1.152	0.005	1.428	1.413	0.006
**AGAMUR-pt2**	2.81	4	0.081	1.143	0.005	1.418	1.417	0.014
**PORVAU02**	2.81	0	0.117	1.155	0.001	1.425	1.422	0.001
**this work**	2.81	0	0.115	1.151	0.002	1.422	1.420	0.001
**PORVAU**	2.82	0	0.113	1.151	0.002	1.423	1.419	0.001
**ODAFAA**	2.83	0	0.103	1.147	0.003	1.418	1.425	0.006
**AGAMUR01-pt1**	2.83	5	0.051	1.155	0.005	1.426	1.414	0.006
**AGAMUR01-pt2**			0.089	1.152	0.008	1.409	1.420	0.007
**ECEWOZ-pt1.1**	2.84	15	0.150	1.152	0.003	1.421	1.417	0.011
**ECEWOZ-pt1.2**			0.078	1.149	0.005	1.425	1.415	0.008
**LOCHIU**	2.84	0	0.101	1.110	0.050	1.490	1.405	0.037
**ECEWOZ-pt2.1**	2.85	3	0.107	1.153	0.004	1.427	1.416	0.004
**ECEWOZ-pt2.2**			0.071	1.155	0.005	1.419	1.416	0.004
**XOKZIG-pt1**	2.85	10	0.116	1.158	0.003	1.451	1.402	0.001
**XOKZIG-pt2**			0.087	1.147	0.004	1.408	1.427	0.008
**YIGVAM**	2.86	0	0.088	1.139	0.001	1.425	1.428	0.004
**NOZZAG**	2.87	0	0.087	1.151	0.001	1.418	1.422	0.002
**XIDVEL**	2.87	0	0.077	1.147	0.004	1.408	1.421	0.006
**QUKNEQ**	2.87	0	0.085	1.148	0.002	1.420	1.418	0.003
**IMEYEE**	2.88	0	0.099	1.145	0.004	1.428	1.422	0.008
**MEFECN**	2.90	0	0.073	1.128	0.015	1.353	1.460	0.011
**MIKTEF**	2.90	0	0.103	1.110	0.010	1.372	1.449	0.025

**KAXLEA-pt1**	2.91	0	0.101	1.141	0.006	1.436	1.416	0.009
**KAXLEA-pt2**		0	0.075	1.139	0.002	1.436	1.413	0.006
**XEYQEZ**	2.91	0	0.067	1.133	0.001	1.451	1.416	0.001
**QIVROD-pt1**	2.91	0	0.066	1.150	0.003	1.421	1.421	0.003
**YAZNUI**	2.92	0	0.076	1.140	0.007	1.397	1.431	0.005
**RIFQAY02-pt1**	2.92	0	0.066	1.148	0.016	1.385	1.436	0.014
**LOCHEQ-pt1**	2.93	11	0.050	1.148	0.003	1.394	1.421	0.008
**LOCHEQ-pt2**			0.088	1.149	0.003	1.406	1.411	0.005
**RIFQAY02-pt2**	2.93	0	0.068	1.153	0.007	1.426	1.418	0.010
**BEPFIN**	2.93	0	0.063	1.146	0.001	1.539	1.390	0.001
**MIKTAB**	2.93	0	0.119	1.132	0.013	1.459	1.418	0.022
**KUGHUP**	2.96	0	0.021	1.157	0.003	1.423	1.418	0.002
**QIVROD-pt2**	2.97	0	0.087	1.144	0.004	1.418	1.419	0.008
**KIYHIJ**	2.99	0	0.047	1.140	0.001	1.420	1.421	0.002
**ECEWUF**	3.00	0	0.068	1.137	0.012	1.409	1.414	0.014
**KAXLIE**	3.09	0	0.057	1.101	0.039	1.445	1.406	0.064
**BUHPIF-pt1**	3.04	113	0.044	1.153	0.002	1.421	1.416	0.003
**BUHPIF-pt2**			0.033	1.153	0.004	1.424	1.416	0.008
**RIFQAY**	3.47	0	0.049	1.145	0.009	1.400	1.423	0.009
**KATJOE**	3.50	0	0.030	1.146	0.020	1.373	1.435	0.021
**RIFQAY01**	3.52	0	0.063	1.142	0.004	1.397	1.426	0.009
**SEJHIY**	4.34	0	0.004	1.124	0.003	1.366	1.445	0.001
**ZOYROU**	4.38	0	0.213	1.133	0.005	1.459	1.433	0.005
**JACSEL**	4.41	0	0.023	1.140	0.005	1.360	1.426	0.018
**UBEJEQ**	4.45	0	0.219	1.133	0.012	1.484	1.447	0.020
**RAZBUP**	5.43	0	0.299	1.141	0.005	1.529	1.445	0.012
**VIBYUB**	4.84	0	0.221	1.143	0.004	1.479	1.441	0.005
**VIBZAI**	5.21	0	0.224	1.143	0.006	1.478	1.444	0.004
**TEFMUM**	6.55	4	0.037	1.093	0.021	1.400	1.468	0.053
